# Body Potassium Content and Radiation Dose from ^40^K for the Urals Population (Russia)

**DOI:** 10.1371/journal.pone.0154266

**Published:** 2016-04-25

**Authors:** Evgenia I. Tolstykh, Marina O. Degteva, Nikolay G. Bougrov, Bruce A. Napier

**Affiliations:** 1 Biophysics Laboratory, Urals Research Center for Radiation Medicine, Chelyabinsk, Russia; 2 Pacific Northwest National Laboratory Richland, Washington, United States of America; North Shore Long Island Jewish Health System, UNITED STATES

## Abstract

Long-term whole-body monitoring of radionuclides in residents of the Urals Region has been performed at the Urals Research Center for Radiation Medicine (URCRM, Chelyabinsk). Quantification of ^40^K was achieved by measuring the ^40^K photopeak with four phoswich detectors in whole body counter SICH-9.1M. The current study presents the results of ^40^K measurements in 3,651 women and 1,961 *t*-test; *U*-test men aged 11–90; measurements were performed in 2006–2014. The residents belonged to two ethnic groups, Turkic (Tatar, Bashkir) and Slavs (mainly Russian). The levels of ^40^K-body contents depend upon gender, age, and body mass. Significant ethnic-differences were not found in ^40^K-body contents and ^40^K concentrations in terms of Bq per kg of body weight (in groups homogenous by age and gender). Both ^40^K-body contents and concentrations were significantly higher in men than in women in all age-groups; the difference was about 25%. The measured ^40^K-body content in men of 20–50 years was about 4200 Bq (134 g of K) and about 3000 Bq (95 g of K) in women. By the age of 80 these values decreased to 3200 Bq (102 g of K) in men and 2500 Bq (80 g of K) in women. Annual dose rates were maximal in the age group of 20–30 years– 0.16 mGy/y for men and 0.13 mGy/y for women. Further, the dose-rates decreased with age and in the groups of 60–80 years were 0.13 mGy/y for men and 0.10 mGy/y for women. Within groups homogeneous by age and gender, individual dose rates are described by a normal statistical distribution. The coefficient of variation ranges from 9 to 14%, and on the average is 12.5%. Doses from naturally occurring ^40^K accumulated over 70 years were found to be 9.9 mGy for men and 8.3 mGy for women; over 90 years - 12.5 and 10.4 mGy.

## Introduction

Potassium-40 is a naturally occurring radionuclide. According to various data its content in the body of an adult person with body weight 70 kg ranges from 4,000 to 5,000 Bq [1] due to the considerable amount of stable potassium in the body (about 140 g). Because the relative content of ^40^K in a mixture of natural potassium isotopes is rather constant, whole body counter measurements allow for the assessment of the potassium body content on the basis of the ^40^K data.

As has been shown [2,3], the major part of the body potassium is accumulated in muscle tissue; the amount of potassium in adipose and bone tissue is relatively small. As a result, the amount of potassium in the body is proportionate to the muscle mass, which in its turn depends on sex, age, and physical activity level. Muscle mass also depends on human ethnicity predetermining the main constitutional features, i.e., percentage of muscle mass, height, and body weight [2–5]. In the period 1974–1997 extensive measurements of the radionuclide body content in Urals residents were performed with the use of Whole Body Counter (WBC) SICH-9.1 created at URCRM [6]. This WBC was designed to measure the body content of ^90^Sr, ^137^Cs and, ^40^K in Urals residents, whose diet was contaminated with radioisotopes after radiation accidents. The accidents occurred in the 1950s [7] due to activity of the “Mayak” Production Association—the first Russian plant for weapons-grade plutonium production. In recent years, i.e., from 2006 and up to the present time, the measurements were performed using the updated WBC SICH-9.1M [8,9]. As a result, a significant data set on ^40^K body content for persons of different age and gender were accumulated at URCRM, and this allows estimation of the amount of body potassium and doses from ^40^K. The comparison of ^40^K body content measured with SICH-9.1 and SICH-9.1M showed that for persons with similar body characteristics (gender, age, body mass, and height) both SICH WBCs had detected similar values of ^40^K in the body [10]. The specific tasks of the study were the following:

1. To analyze the results of the ^40^K body content measurements in Urals residents of different age, gender, ethnicity and anthropometric parameters, obtained in 2006–2014.
2. To calculate the annual absorbed dose rates in persons of different age, gender, and also the doses accumulated during life.

## Materials and Methods

### Ethics Statement

The study was approved by the ethics committee of Urals Research Center for Radiation Medicine (URCRM), Chelyabinsk, Russia. All individuals signed a written informed consent to medical examination in URCRM which include WBC measurements; parents or guardians gave written consent on behalf of children before 18 years; they also signed a written consent to processing of personal data.

### Measurement of ^40^K with use the whole body counter SICH-9.1M

Specific characteristics of whole body counter SICH-9.1M, its construction and operation have been described previously [8,9]. The measurements were performed with use of four phoswich detectors (Saint-Gobain Cristaux&Detecteurs, France) in a specially shielded room equipped with scanning-bed geometry. Analyses of ^40^K were performed by measurement of the photopeak. A solid whole-body phantom set assembled of right-angled polyethylene units and rod sources of ^40^K inserted into them was used for calibration for ^40^K. This phantom set (UP-02T) simulates the body characteristics of children and of adults of different weights [11]; rod sources containing standard activities of ^40^K are inserted into the phantom blocks. For the measurement period 2006–2014 the mean value of the detection limit for ^40^K was 345 Bq; the mean value of the minimum detectable activity was 1,035 Bq. Taking into account ^40^K natural abundance of 0.0118% and specific activity of 2.652× 10^5^ Bq/g, the value of natural potassium specific activity of 31.29 Bq g^-1^ (2.652·10^5^ × 1.18·10^−4^) was used to estimate the body potassium content.

### Characteristics of the Urals residents measured with SICH-9.1M

The current study is based on SICH-9.1M measurements performed over the period from June 2006 to November 2014: 3,651 measurements of women; 1,961 measurements of men; 5,612 measurements in total. Table 1 shows the age and gender characteristics of measured persons. Two primary ethnic groups have been investigated: (1) Turks—mainly Tartars, and also Bashkirs; (2) Slavs- mainly Russians, and also Ukrainians, Byelorussians. According to our data obtained in 1974–1997, Slavic and Turkic peoples statistically significantly differ in terms of the anthropometric parameters (height and body weight). Measurements of weight and height were performed with standard methods before the WBC measurements. Body mass index was calculated as the ratio of body weight (kg) to square of body height (m^2^). As can be seen from Table 1, the differences in height and weight between these ethnic groups are manifest only for elder age groups, both for men and women; up to the age of 40 these differences are not observed. The age and gender composition of measured persons reflects the age-structure of people undergoing medical examination at URCRM in relation to the radioactive contamination of the Urals region in 1950s.

**Table 1:** Anthropometric characteristic of persons under study.

Age range	Age, М±STDV	Number of subjects	Height, mm М±STDV	Weight, kgМ±STDV	Body-mass index, M±STDV
			**Slavic men**		
**12–19**	16±2.3	10	1668±113	55.3±15	19.5±3.0
**20–29**	25±2.8	36	1738±66	75.4±18	24.9±5.1
**30–39**	35±3.1	54	1757±55	82.7±17	26.8±5.2
**40–49**	45±2.9	76	1753±55	84.6±16*	27.5±4.9
**50–59**	55±3.0	209	1708±54*	81.8±15	28.0±4.7
**60–69**	64±3.0	215	1694±65*	79.9±15*	27.7±4.5
**70–79**	73±2.6	142	1659±60*	77.6±15*	28.2±5.1
**>80**	81±1.5	13	1635±46*	68.7±10	25.8±3.8
			**Turkic men**		
**12–19**	17.0±2.2	21	1701±84	62.4±11.7	21.5±3.0
**20–29**	24.2±3.0	67	1720±71	69.5±13.6	23.4±4.1
**30–39**	35.4±3.1	84	1713±60	78.2±13.4	26.7±4.6
**40–49**	45.4±2.7	196	1692±67	77.1±14.8*	26.9±4.5
**50–59**	54.6±2.9	368	1686±57*	80.9±14.4	28.4±4.6
**60–69**	64.2±3.2	275	1667±62*	77.8±14.4*	27.9±4.4
**70–79**	73.2±2.7	178	1624±54*	73.1±11.1*	27.7±3.9
**>80**	82.4±2.3	17	1630±42*	70.7±10.9	26.6±4.1
			**Slavic women**		
**11–19**	16.9±2.6	10	1614±61	54.9±12.3	21.0±4.2
**20–29**	24.6±3.1	48	1607±63	60.6±14.9	23.5±5.7
**30–39**	35.1±2.8	80	1617±59	68.7±14.6	26.3±5.7
**40–49**	45.1±2.8	129	1608±60*	75.8±15.1	29.4±5.9
**50–59**	55.0±2.9	417	1580±53*	79.4±16.2*	31.8±6.6
**60–69**	64.2±3.1	389	1562±53*	77.3±16.0	31.6±6.1
**70–79**	73.3±2.7	291	1530±53*	73.9±14.3	31.5±5.7
**>80**	82.5±2.9	32	1488±38*	66.1±11.6	29.8±4.7
			**Turkic women**		
**11–19**	16.8±2.2	18	1587±55	55.9±12.4	22.1±4.3
**20–29**	24.7±2.8	78	1608±57	57.1±10.3	22.1±4.0
**30–39**	35.0±2.8	136	1598±57	67.1±14.4	26.3±5.7
**40–49**	45.5±2.7	417	1574±59*	72.8±14.2	29.4±5.7
**50–59**	54.3±2.8	716	1560±53*	74.5±14.1*	30.6±5.6
**60–69**	64.4±3.0	518	1537±53*	76.5±13.9	32.4±5.6
**70–79**	72.9±2.4	349	1512±51*	72.4±13.0	31.6±5.3
**>80**	82.7±2.5	23	1508±52*	67.5±13.3	29.6±4.8

М±STDV—mean±standard deviation;

*- statistically significant difference between ethnic groups of the same age and gender (p<0.05 *t*-test; *U*-test)

It should be noted that the number of examined women was about twice that of men. A significant part of the studied group is older than 50. Confidentiality of the subjects is ensured by restricting access to the identifying personal information only to scientists from the URCRM; for persons from other organizations participating in the studies of this cohort, only the identification code (IC) is available.

### Calculation of internal doses from ^40^K

The Dose Factors (DF, synonym S-values, i.e. energy absorbed per unit mass of target region due to nuclear transformation of ^40^K in source region per sec) described by Strom et al. [12] were as the basis for the calculation of annual dose rate from natural ^40^K (Table 2). These DF were estimated on the basis of RADAR phantoms [13] developed for children of different ages and for adult males and females. The DF were calculated for ^40^K uniformly distributed in the whole body (the whole body is both the source and the target of the exposure). Strom et al. [12] note that the phantoms used [13] did not incorporate a variable body mass and recommended that such a correction be considered. Because a significant portion of the studied Urals group does not correspond to the anthropometric characteristics of the phantoms, the linear weight-scaling recommended by Siegel and Stabin [14] for radionuclides with low penetrating-to-nonpenetrating ratio was applied. Linear weight scaling with the multiplicative factor (W_ph_/W_ind_) was used to arrive at the appropriate DF-value for an individual patient:

$$DF_{ind}=DF_{ph}\frac{\;W_{ph}}{{\text{W}}_{ind}}\;;$$ (1)

Where:

*DF*_*ind*_ = dose factor corrected on individual weight;

*DF*_*ph*_ = dose factor basing on phantom estimates;

*W*_*ph*_ = weight of phantom, kg.

*W*_*ind*_ = individual patient weight, kg

**Table 2:** Characteristics of phantoms, DF-values and dose coefficients for ^40^K derived from [12].

Phantom	DF-value ×10^-12^ mGy·Bq^-1^·s^-1^	Phantom weight, kg	DC ×10^−12^ mGy·s^-1^ (Bq kg^-1^)^-1^	DC mGy year^-1^ (Bq kg^-1^)^-1^	Patient weight, kg
Adult male	1.2	73.7	88.44	0.002791	65–85
Adult female	1.54	56.8	87.47	0.002760	45–65
15 years	1.54	56.8	87.47	0.002760	45–65
10 years	2.61	33.2	86.65	0.002734	30–45
5 years	4.35	19.8	86.13	0.002718	-
1 year	8.76	9.72	85.15	0.002687	-
New-born	23.3	3.6	83.88	0.002647	-

For technical convenience of individual dose calculation, the *DF*_*ph*_ and *W*_*ph*_ were multiplied to receive the Dose Coefficient (DC) for each phantom used. Therefore, the annual dose rate from natural ^40^K (*D*_*ind*_) was calculated as:

$$D_{ind}=DC\frac{\;A_{ind}}{{\text{W}}_{ind}}$$ (2)

Where:

*A*_*ind*_ = individual body activity measured with SICH-9.1M;

Table 2 presents two sets of values: (1) the original values of DF in terms of mGy per sec per 1 Bq of ^40^K (energy absorbed in 1 kg of the phantom (target) due to one decay per second of ^40^K in the whole phantom (source)); and (2) calculated DCs in terms of Gy per sec per 1 Bq of ^40^K in 1 kg of phantom (energy absorbed in 1 kg of the phantom (target) due to one decay per second of ^40^K in 1 kg of phantom (source)). As can be seen from Table 2, the differences between DCs for different phantoms are minor and result because of the slightly increased escape of ^40^K photons from the smaller bodies of children and females than from adult males.

**Fig 1:**
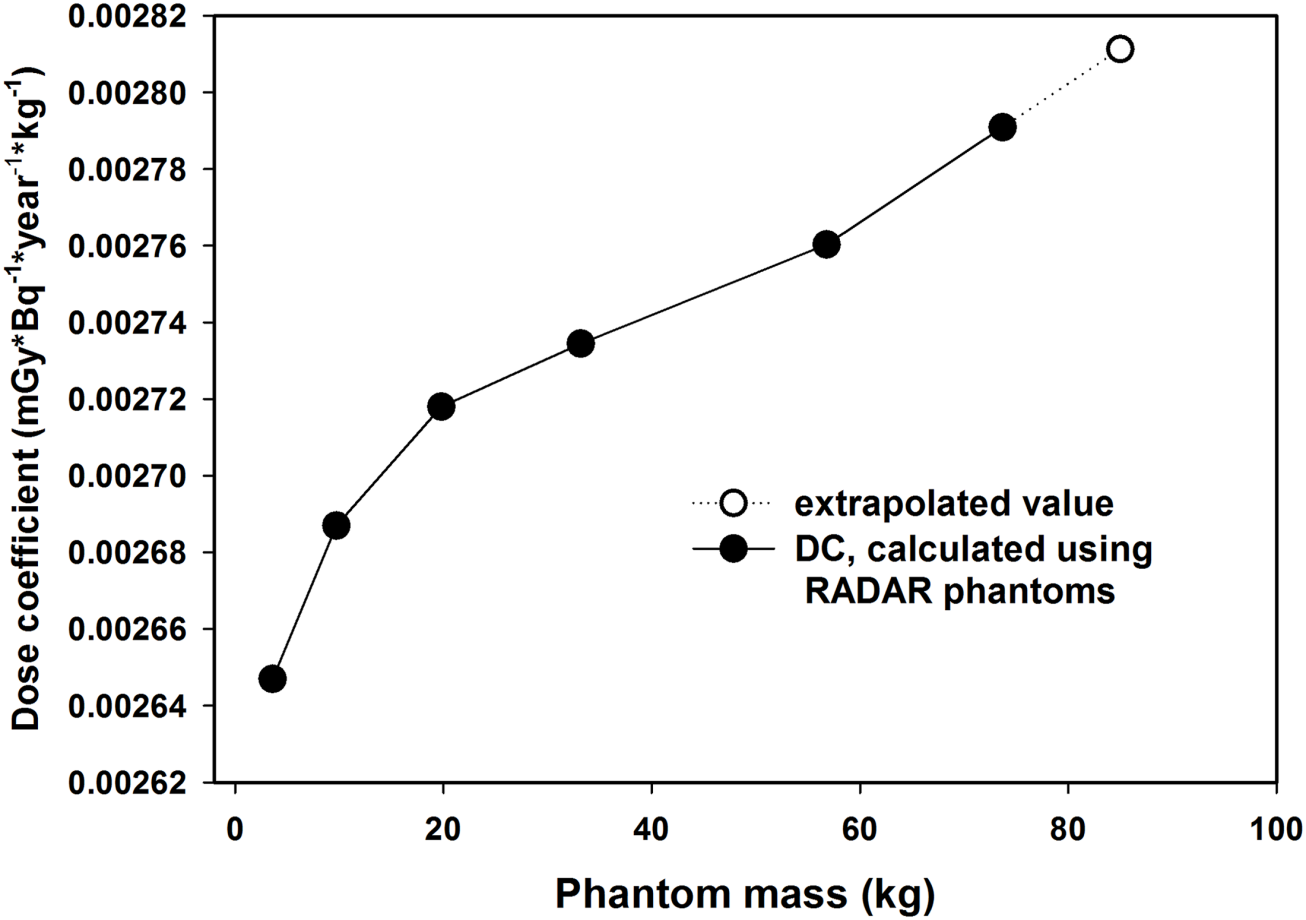
Dependence of dose coefficient on body weight.

It should be noted that the body weight of a considerable number of measured Urals persons exceeded the standard 73.7 kg. Thus, we have performed a linear extrapolation of the DC for a body weight of 73.7 kg to a body weight of 85 kg. Fig 1 illustrates the principle of the extrapolation. The obtained coefficient (88.71×10^−12^ mGy·s^-1^ (Bq kg^-1^)^-1^) was used for subjects with body weight of 85 kg and more.

### Statistical methods

Standard statistical methods were used in the current study. The significance of the differences between the groups was assessed using the Student t-test and Mann Whitney U-test because height, weight, and ^40^K body content within the groups homogeneous in gender and age were mostly described with a normal distribution, and for a few groups described with non-normal distribution. Differences were assumed to be statistically significant when p<0.05. Distributions were checked for normality using the Shapiro-Wilk test, statistically significant values of W-test (p<0.05) evidenced that the distribution was not normal. Correlation analysis and multiple regression analysis (weight estimates) were used for estimating the relationship between anthropometric characteristics, age and ^40^K body-content, and for estimation of the relative effect of BMI and age on the ^40^K variability in patients over 40. For the calculations licensed software Microsoft Excel 2010 and Sigma Plot 12.0 was used.

## Results

### Total body potassium and ^40^K body content in Urals residents depending on the gender, age and ethnicity

**Fig 2:**
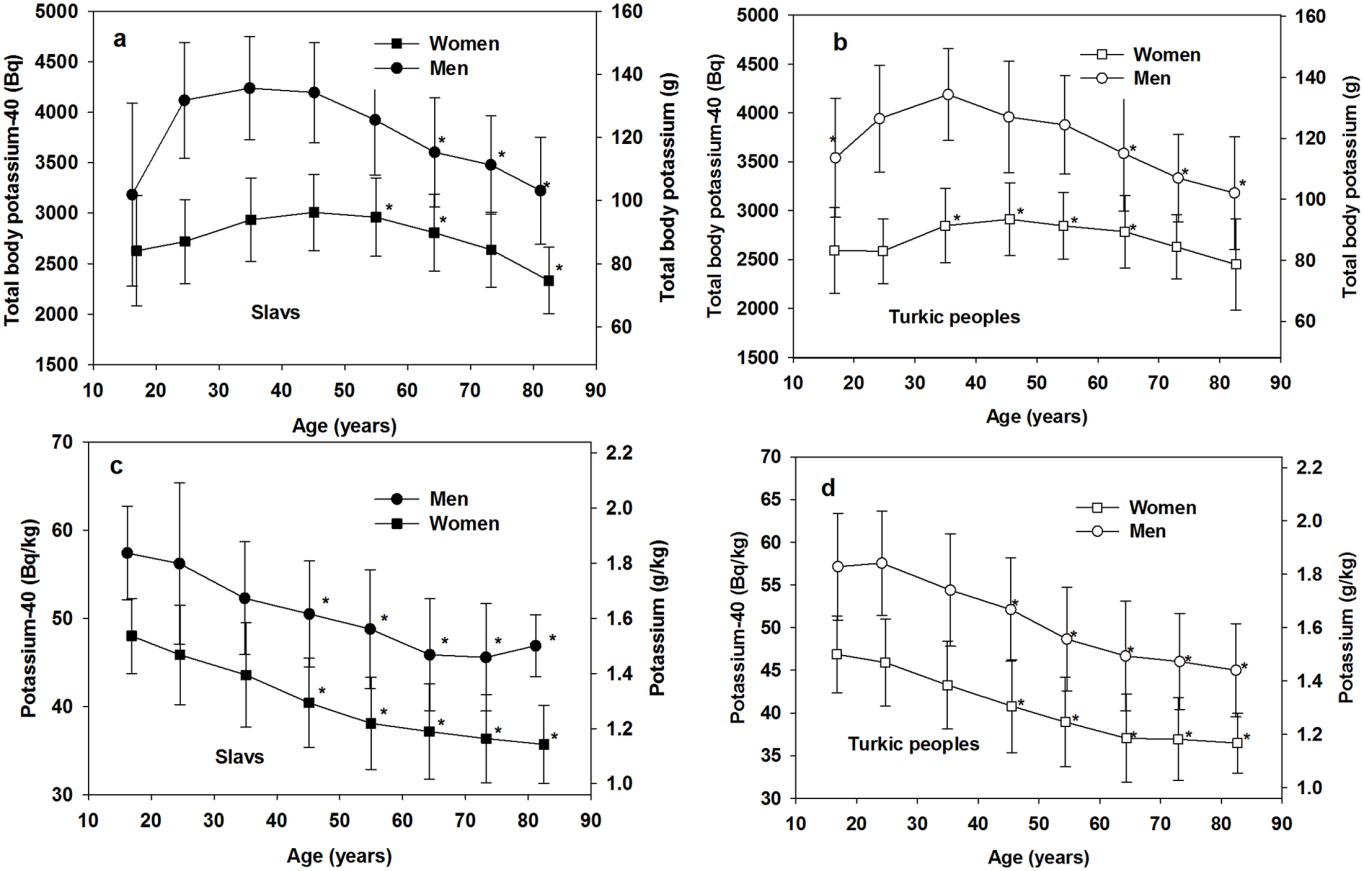
^40^K and stable potassium body contends in men and women depending on age gender and ethnicity. (a) Slavs, total body potassium and ^40^K; (b) Turkic peoples, total body potassium and ^40^K; (c) Slavs, body potassium g/kg and ^40^K Bq/kg; (d) Turkic peoples, body potassium g/kg and ^40^K Bq/kg. Bars represent the range of standard deviation. *—statistically significant differences from the value for the age group 20–30 of the respective gender (*t*-test; *U*-test).

The results of ^40^K body measurements are presented in Fig 2. As can be seen from Fig 2, there are similar age-dependences of the ^40^K body content for men and women. Maximal ^40^K body content was observed for persons aged from 20 to 40–50; after that the ^40^K body content gradually decreased. ^40^K body content in terms of Bq/kg also decreased after the age of 40 both in men and women of either ethnic group. In all age groups the ^40^K body content is about 25% higher in men than in women. This can be explained by the higher percentage of muscle mass in men relative to women [15] and lower BMI (Table 1). The comparison of the ^40^K body content between ethnic groups did not demonstrate statistically significant differences with the exception of the group of men aged 40–50 and the group of women aged 50–60. Similarly, there were no statistically significant differences in the concentration of ^40^K in the body of Slavs and Turkic people. Thus, the homogeneous in terms of gender and age groups of Slavs and Turkic people were combined for further analysis.

The total body potassium and concentration, age and BMI are usually considered as related parameters. Body mass index characterizes the level of obesity, and increased BMI is associated with a decreased percentage of muscle body mass (main potassium depot) and increased fat mass. Thus, potassium concentrations were inversely correlated with body mass index [16]. Table 3 shows the Pearson correlation coefficients for the studied parameters. The non-parametric Spearman correlation coefficients were also estimated. Because they were similar with Pearson coefficients (Table 3), the Table of Spearmen correlation coefficients was included as a supporting document. It should be noted that the age-related changes of the muscular system (depot of potassium), adipose and skeletal system differ considerably in men and women due to different dynamics of sex-steroid production affecting the skeletal and muscle masses. Sharp decrease in sex steroids production after menopause is observed in women; as for men, gradual age—dependent decline is observed after the age of 55. Correlation analysis was performed for men and women separately; three age groups were selected for analysis (Table 3).

**Table 3:** Pearson correlation coefficients for ^40^K characteristics, age and BMI in different age- groups of men and women.

Women				Men		
	^**40**^**K (Bq/kg)**	**BMI**	**Age (25–40 y)**	^**40**^**K (Bq/kg)**	**BMI**	**Age (25–40 y)**
^**40**^**K (Bq)**	-0.15*	0.71	0.20	-0.14*	0.70	0.13**
^**40**^**K (Bq/kg)**	-	-0.70	-0.27	-	-0.70	-0.18
**BMI**	-	-	0.31	-	-	0.22
	^**40**^**K (Bq/kg)**	**BMI**	**Age (40–80 y)**	^**40**^**K (Bq/kg)**	**BMI**	**Age (40–80 y)**
^**40**^**K (Bq)**	0.04*	0.57	-0.3	0.20	0.59	-0.42
^**40**^**K (Bq/kg)**	-	-0.70	-0.26	-	-0.54	-0.32
**BMI**	-	-	0.11	-	-	0.03**
	^**40**^**K (Bq/kg)**	**BMI**	**Age (60–80 y)**	^**40**^**K (Bq/kg)**	**BMI**	**Age (60–80 y)**
^**40**^**K (Bq)**	0.03**	0.62	-0.31	0.21	0.60	-0.28
^**40**^**K (Bq/kg)**	-	-0.65	-0.09	-	-0.50	-0.10
**BMI**	-	-	-0.07*	-	-	-0.025**

Comments: in all cases (excepting indicated by *) p<0.001;

*- p<0.05;

**- p>0.05; there is no significant relationship between the two variables.

In 25–40 group n = 313 (women) and n = 203 (men); in 40–80 group n = 3281 (women) and n = 1689 (men); in 60–80 group n = 1602 (women) and n = 840 (men)

#### Age-group 25–40

The age 25–40 is the period of stable body height, relatively stable body weight, and of slightly changing body potassium content. The relationship between the studied parameters are the same for men and women. According to Table 3, there is some age-dependent increase in whole body potassium content (the correlation is significant for women) due to the increase in body weight, resulting in the increase in BMI (significantly for both men and women). At the same time the potassium concentration decreases with age (significantly both in men and women) as age-dependent weight gain usually occurs as a result of adipose tissue, i.e. content of the muscle tissue (potassium depot) decreases. This fact explains the negative correlation between BMI and the ^40^K concentration. The significant (but weak) negative relationship between ^40^K body content and ^40^K concentration also suggests that increase in body mass and BMI occurs due to the gain of fat tissue with very small potassium content.

#### Age-group 40–80

The age 40–80 is a period of significant age-related changes in body weight, height, and percentage of muscle mass. The positive correlation between age and total-body-potassium (characteristic of the previous age period) becomes negative. This indicates age-dependent decline of absolute muscle mass. Continuing increase of the BMI with age (significant in women) testifies not only to additional gain of adipose tissue, but also to the decrease in body height with age (Table 1). Positive relationship between ^40^K content and ^40^K concentration observed in men and women serves as additional prove of this fact. The relationship between BMI and the potassium content/concentration remains negative.

Relative contribution of age and BMI to the changes of ^40^K concentration was evaluated with the use of multiple linear regression and weight estimates of each variable (Table 4). Regression parameters were different for men and women, but the general patterns were similar: BMI and age together, as independent variables, explain about half (40% for women and 50% for men) of the variability of the dependent variable (^40^K concentration); both variables statistically significantly predict the dependent variable, ^40^K concentration (*p*<0.001). The contribution of variable “BMI” is greater than that of “age”: for male the relative contribution of BMI is 2-times higher than that of age (0.56 and 0.29); for women—it is 3-times higher (0.68 and 0.18).

**Table 4:** Parameters of multiple regressions for two independent variables (age, BMI) which predict specific activity of ^40^K.

Group of those aged 40–80	Coefficient of multiple correlation R^2^	Standardized Beta 1 (variable Аge)	Standardized Beta 2 (variable BMI)
**Male**	0.402 (p<0.001; F = 568)	-0.293±0.019 (p<0.001)	-0.559±0.019 (p<0.001)
**Female**	0.516 (p<0.001; F = 1745)	-0.180±0.012 (p<0.001)	-0.675±0.012 (p<0.001)

#### Age-group 60–80

The age period 60–80 was extracted from the previous age-group; this period is characterized by pronounced senile changes in the skeletal and muscular system. A weak negative correlation between age and body mass index is observed only in this period. All the other dependences are the same as for the whole age group 40–80.

### Absorbed doses from ^40^K in the human body

Individual annual absorbed dose rates for all persons under study were calculated with use of DCs described above (Table 2). Table 5 presents the description of the results. According to the data from Table 5, annual dose rates in men and women differ in all age groups. The difference was about 25%.

**Table 5:** Characteristics of annual dose rates for Urals residents depending on gender and age.

Age group, years	N	Annual dose rate, mGy М±STDV	CV%	Shapiro-Wilk test	
				W-test	P
		**Women**			
**15–19**	28	0.131±0.012	9	0.973	*0*.*668*
**20–29**	142	0.126±0.014	11	0.984	*0*.*099*
**30–39**	200	0.120±0.015^a^^b^	12	0.996	*0*.*931*
**40–49**	546	0.113±0.014 ^a^^b^	13	0.996	*0*.*123*
**50–59**	1133	0.108±0.014 ^a^^b^	13	0.998	*0*.*188*
**60–69**	907	0.103±0.014 ^a^^b^	14	0.990	<0.001*
**70–79**	640	0.102±0.013^a^	13	0.992	0.002*
**>80**	55	0.100±0.011^a^	11	0.984	*0*.*677*
		**Men**			
**15–19**	35	0.157±0.016	10	0.955	*0*.*157*
**20–29**	103	0.159±0.020	12	0.965	0.008*
**30–39**	138	0.150±0.018 ^a^^b^	12	0.991	*0*.*550*
**40–49**	272	0.144±0.016 ^a^^b^	11	0.994	*0*.*290*
**50–59**	577	0.136±0.017 ^a^^b^	13	0.997	*0*.*520*
**60–69**	490	0.129±0.017 ^a^^b^	13	0.960	< 0.001*
**70–79**	320	0.128±0.016^a^	12	0.992	*0*.*098*
**>80**	30	0.127±0.013^a^	10	0.962	*0*.*357*

^a^- statistically significant differences with the age group 20–29 of the respective sex (*t*-test; *U*-test);

^b^- statistically significant differences with preceding age group (*t*-test; *U*-test),

*—W-test is statistically significant; hypothesis about normal distribution of the values of the variable is rejected, data are not described by normal distribution. In other cases data correspond to normal distribution.

Annual dose rates were maximal in age groups up to the age of 30: 0.13 mGy/y for women and 0.16 mGy/year for men. This corresponds to age-related features of ^40^K body accumulation. Individual values of the dose rates are described with normal statistical distribution except for 2 groups of women (aged 60–69 and 70–79) and of men (aged 20–29 and 60–69). Coefficient of variation ranges from 9 to 14%, on the average– 12.5%. The obtained values (Table 5) are lower than reported by UNSCEAR [17]: 0.165 and 0.185 mSv/y for adults and children, respectively. The main reasons could be the use of different calculation approaches, phantoms and estimated specific absorption fractions.

For some risk assessment studies it is important to estimate the ^40^K doses accumulated during the human life. However, the annual dose rates are not available for Urals children under the age of 15 (Table 5), because only a few measurements of teenagers aged 9–14 were performed, and persons under the age of nine were not measured at all.

The changes in potassium body content during childhood are mainly determined by the changes in tissue composition (percentage of muscle mass). As has been shown [1, 18, 19], the concentrations of potassium in the body was about 15–20% higher in boys than in girls in teenagers aged 11–12, but as whole the values were lower than that in young adult men of 30 years. In a younger group (aged 5–10) the concentration of ^40^K in the body did not differ significantly in boys and girls, and the value was close to that in girls aged 11–12. The number of ^40^K measurements in children younger than 5 years old is very limited and mainly related with newborns and infants before 24 months [20–22]. Measured values were lower than that in children of 5–10 years but not lower than in adult women of 40–50 [1]. Taking into account these age-dependences, the following ^40^K concentration in children and teenagers were assumed for calculations: 10–14 years, men– 55 Bq/kg, women– 47 Bq/kg; 5–10 year– 45 Bq/kg; 0–5 years—42 Bq/kg. Thus, the absorbed accumulated doses from ^40^K obtained with use of the DC from Table 2 were as follows:

Men: over 70 years old– 9.9 mGy; over 90 years– 12.5 mGy.
Women: over 70 years old– 8.3 mGy; over 90 years– 10.4 mGy.

## Discussion

Table 6 exemplifies the published data on potassium measurements (professionals-workers of nuclear production plants and non-professional persons) in comparison with Urals data obtained in current study.

**Table 6:** Comparison of the results of ^40^K measurements for adult persons with published data.

Group	^40^K kBq		K g/kg		Source of information
	Women	Men	Women	Men	
**Hanford workers**	3.1±0.2; n = 248	4.2±0.1; n = 2037	1.4±0.3	1.7±0.3	Lynch et al [16] ^a^
**Egyptian radiation workers**	4.1±0.3; n = 47	5.2±0.2; n = 192	2.0±0.5	2.1±0.4	Gohary et al [23]
**US residents (White)**	2.9±0.4; n = 669	4.7±0.7; n = 444	1.5±0.2	1.9±0.3	He et al [5] ^b^
**US residents (Asian)**	2.6±0.3; n = 197	4.1±0.4; n = 152	1.5±0.2	1.9±0.3	
**Urals residents** ^c^	2.8±0.4; n = 313	4.2±0.5; n = 203	1.4±0.2	1.7±0.2	Current study

^a^- similar results were presented in [12] Strom et al., 2009 based on measurements performed in 2006–2007

^b^- data were obtained from ^40^K measurements; original values presented in mmol were recalculated in compatible units of K and ^40^K (1 mmol = 39 g potassium; 1 g natural potassium contains 31.29 Bq ^40^K)

^c^- data for persons aged 25–40 years were used

As can be seen, the data on ^40^K body content for the Urals residents do not contradict the published data. They are very close to data on Hanford workers [12, 16] and data on White and Asian US residents [5] and Egyptian male workers [23]. Small differences between samples can be explained by differences in anthropometric characteristics. Significant differences are observed for Egyptian women-workers that can be explained by the features of the sample (small number of persons or others).

Data on ^40^K body content are used for estimates of skeletal muscle mass; the value of muscle mass can be obtained by multiplying by a conversion factor [18]. Therefore, the relationship of ^40^K content and body weight, age and BMI is interpreted as a correlation for muscle mass. Age-dependent decrease in muscular mass is a serious health problem. According to our data standardized body mass and age together explain only 40–50% of ^40^K-concentration variability at the age of 40–80; and age (i.e. age-related set of physiological parameters) has lower impact on ^40^K-concentration than BMI. According to the published data [24–27], in elderly persons decrease in muscle mass (so called sarcopenia) is associated with a significant number of physiological reasons: changes in hormonal levels including decrease in the concentrations of growth hormone, testosterone, and insulin-like growth factor-I; loss of motor neurons and the rate of motor units remodeling; increase in oxidative damage levels; decrease of protein synthesis; inflammatory process; lifestyle factors, etc. As it has been recently shown, older women with sarcopenia (decreased muscle mass and therefore decreased total body potassium) have an increased all-cause mortality risk independent of obesity [28].

Therefore, the results of our studies could be applied to two scientific areas: (1) dose estimates from naturally occurring radionuclides and their uncertainties, and (2) support of the future study of age-changes in skeletal muscles and age-related diseases (cardiovascular, metabolic diseases, and others) in the Urals population. The URCRM databases containing the individual medical information on studied persons provide the basis for such investigations.

## Conclusions

Total ^40^K content and concentration are considerably higher in men as compared to those in women in the studied age range; the differences were about 25%. No statistically significant differences in ^40^K content and concentration were revealed between the two ethnic groups (Slavs and Turkic people). Maximal ^40^K body-content was observed for persons aged from 20 to 40–50. After that age a gradual decrease of ^40^K body content occurs. A significant negative correlation was found between ^40^K body concentration and two important parameters: BMI (characterizing the level of obesity) and age.

Annual dose rates were maximal in the age group 20–30: 0.13 mGy per year for women and 0.16 mGy per year for men, which reflect the age peculiarities of ^40^K body content. Within groups homogeneous in sex and age individual values of dose rate are described with a normal statistical distribution. The coefficient of variation ranges from 9 to 14%, on the average– 12.5%. The internal dose from naturally occurring ^40^K accumulated over 70 years was 9.9 mGy for men and 8.3 mGy for women.

## Supporting Information

S1 Table Characteristics of ^40^K body content for men and women of different gender, age and ethnicity. (PDF)

S2 Table Characteristics of ^40^K body concentration for men and women of different gender, age and ethnicity. (PDF)

S3 Table Spearman correlation coefficients for ^40^K characteristics, age, and BMI in different age- groups of men and women. (PDF)

S4 Table Median values and quartile range for ^40^K annual dose rate for men and women. (PDF)
