# Supplementary material for: Body Potassium Content and Radiation Dose from 40K for the Urals Population (Russia)
Source: PLoS One. 2016 Apr 25;11(4):e0154266. doi: 10.1371/journal.pone.0154266 (PMC4844139; doi:10.1371/journal.pone.0154266)
Supplement: S1 Table — (PDF) [file pone.0154266.s001.pdf]

S1 Table. Characteristics of ^{40}K body content for men and women of different gender, age and ethnicity

Age group, years	N	^{40}K body content, Bq M±STDV	CV%	Shapiro-Wilk test	
				W-test	P
**Turkic men**					
15-19	21	3541 ±609	17	0.959	0.498
20-29	67	3942 ±546	14	0.982	0.436
30-39	84	4189 ±468	11	0.987	0.592
40-49	196	3958 ±572	14	0.987	0.068
50-59	368	3879 ±505	13	0.992	0.043*
60-69	275	3587 ±588	16	0.959	<0.001*
70-79	178	3334 ±445	13	0.992	0.397
>80	17	3182 ±577	18	0.956	0.55
**Slavic men**					
15-19	10	3184 ±909	29	0.899	0.213
20-29	36	4119 ±575	14	0.963	0.269
30-39	54	4239 ±511	12	0.957	0.05
40-49	76	4196 ±497	12	0.979	0.237
50-59	209	3925 ±549	14	0.99	0.162
60-69	215	3604 ±540	15	0.995	0.722
70-79	142	3479 ±486	14	0.987	0.193
>80	13	3222 ±532	16	0.951	0.614
**Turkic women**					
15-19	18	2592 ±438	17	0.972	0.829
20-29	78	2586 ±331	13	0.99	0.803
30-39	136	2848 ±381	13	0.986	0.198
40-49	417	2913 ±372	13	0.975	<0.001*
50-59	716	2845 ±343	12	0.994	0.009*
60-69	518	2786 ±371	13	0.992	0.008*
70-79	349	2629 ±328	12	0.996	0.51
>80	23	2451 ±467	19	0.966	0.598
**Slavic women**					
15-19	10	2627 ±548	21	0.938	0.534
20-29	48	2719 ±416	15	0.962	0.117
30-39	80	2935 ±415	14	0.976	0.133
40-49	129	3007 ±378	13	0.987	0.265
50-59	417	2960 ±388	13	0.996	0.347
60-69	389	2807 ±381	14	0.996	0.383
70-79	291	2638 ±371	14	0.977	<0.001*
>80	21	3541 ±609	17	0.975	0.639

* - W-test is statistically significant; hypothesis about normal distribution of the values of the variable is rejected, data are not described by normal distribution. In other cases data correspond to normal distribution
