# Supplementary material for: Body Potassium Content and Radiation Dose from 40K for the Urals Population (Russia)
Source: PLoS One. 2016 Apr 25;11(4):e0154266. doi: 10.1371/journal.pone.0154266 (PMC4844139; doi:10.1371/journal.pone.0154266)
Supplement: S2 Table — (PDF) [file pone.0154266.s002.pdf]

S2 Table. Characteristics of ^{40}K body concentration for men and women of different gender, age and ethnicity

Age group, years	N	^{40}K concentration, Bq/kg, M±STDV	CV%	Shapiro-Wilk test	
				W-test	P
**Turkic men**					
15-19	21	57.1 ±6.2	11	0.959	0.49
20-29	67	57.6 ±6.1	11	0.978	0.284
30-39	84	54.4 ±6.6	12	0.986	0.49
40-49	196	52.1 ±6.0	12	0.99	0.169
50-59	368	48.7 ±6.1	12	0.995	0.254
60-69	275	46.7 ±6.4	14	0.921	<0.001*
70-79	178	46.0 ±5.6	12	0.987	0.099
>80	16	44.6 ±5.4	12	0.959	0.637
**Slavic men**					
15-19	10	57.4 ±5.3	9	0.879	0.129
20-29	36	56.2 ±9.1	16	0.955	0.148
30-39	54	52.3 ±6.4	12	0.985	0.709
40-49	76	50.5 ±6.0	12	0.984	0.452
50-59	209	48.8 ±6.7	14	0.989	0.094
60-69	215	45.9 ±6.4	14	0.986	0.033*
70-79	142	45.6 ±6.1	13	0.992	0.561
>80	13	46.9 ±3.5	7	0.961	0.77
**Turkic women**					
15-19	18	46.9 ±4.5	10	0.977	0.912
20-29	78	45.9 ±5.1	11	0.962	0.019*
30-39	136	43.3 ±5.1	12	0.995	0.911
40-49	417	40.8 ±5.4	13	0.995	0.151
50-59	716	38.9 ±5.2	13	0.998	0.362
60-69	518	37.0 ±5.2	14	0.986	<0.001*
70-79	349	36.9 ±4.8	13	0.99	0.018*
>80	23	36.5 ±3.5	10	0.984	0.96
**Slavic women**					
15-19	10	48.0 ±4.2	9	0.983	0.981
20-29	48	45.9 ±5.6	12	0.975	0.379
30-39	80	43.6 ±5.9	14	0.989	0.734
40-49	129	40.4 ±5.0	12	0.992	0.65
50-59	417	38.1 ±5.2	14	0.996	0.377
60-69	389	37.2 ±5.4	14	0.989	0.004*
70-79	291	36.4 ±5.0	14	0.987	0.01*
>80	32	35.7 ±4.4	12	0.976	0.691

* - W-test is statistically significant; hypothesis about normal distribution of the values of the variable is rejected, data are not described by normal distribution. In other cases data correspond to normal distribution
