# Supplementary material for: Body Potassium Content and Radiation Dose from 40K for the Urals Population (Russia)
Source: PLoS One. 2016 Apr 25;11(4):e0154266. doi: 10.1371/journal.pone.0154266 (PMC4844139; doi:10.1371/journal.pone.0154266)
Supplement: S3 Table — (PDF) [file pone.0154266.s003.pdf]

S3 Table. Spearman correlation coefficients for ^{40} K characteristics, age, and BMI in different age- groups of men and women

	Women			Men		
	**^{40}K (Bq/kg)**	**BMI**	**Age (25-40 y)**	**^{40}K (Bq/kg)**	**BMI**	**Age (25-40 y)**
**^{40}K (Bq)**	-0.13*	0.67	0.16	-0.14**	0.67	0.09
**^{40}K (Bq/kg)**	-	-0.69	-0.27	-	-0.68	-0.18
**BMI**	-	-	0.33	-	-	0.21
	**^{40}K (Bq/kg)**	**BMI**	**Age (40-80 y)**	**^{40}K (Bq/kg)**	**BMI**	**Age (40-80 y)**
**^{40}K (Bq)**	0.026**	0.53	-0.29	0.16	0.57	-0.42
**^{40}K (Bq/kg)**	-	-0.71	-0.26	-	-0.55	-0.32
**BMI**	-	-	0.14	-	-	0.03**
	**^{40}K (Bq/kg)**	**BMI**	**Age (60-80 y)**	**^{40}K (Bq/kg)**	**BMI**	**Age (60-80 y)**
**^{40}K (Bq)**	0.013**	0.59	-0.29	0.15	0.61	-0.29
**^{40}K (Bq/kg)**	-	-0.673	-0.08	-	-0.52	-0.10
**BMI**	-	-	-0.041**	-	-	-0.03**

Comments: in all cases (excepting indicated by *) $p<0.001$

*- $p<0.05$;

**- $p>0.05$; there is no significant relationship between the two variables.

In 25-40 group n=313 (women) and n=203 (men); in 40-80 group n=3281 (women) and n=1689 (men); in 60-80 group n=1602 (women) and n=840 (men)
