# Supplementary material for: Body Potassium Content and Radiation Dose from 40K for the Urals Population (Russia)
Source: PLoS One. 2016 Apr 25;11(4):e0154266. doi: 10.1371/journal.pone.0154266 (PMC4844139; doi:10.1371/journal.pone.0154266)
Supplement: S4 Table — (PDF) [file pone.0154266.s004.pdf]

S4 Table. Median values and quartile range for ^{40}K annual dose rate for men and women

Age group	Men			Women		
	Median	25 percentile	75 percentile	Median	25 percentile	75 percentile
10-19	0.157	0.144	0.168	0.131	0.121	0.140
20-29	0.159	0.149	0.173	0.127	0.117	0.138
30-39	0.149	0.139	0.160	0.120	0.110	0.131
40-49	0.143	0.133	0.155	0.113	0.103	0.123
50-59	0.136	0.124	0.147	0.108	0.0980	0.117
60-69	0.130	0.118	0.140	0.102	0.0936	0.113
70-79	0.127	0.118	0.138	0.102	0.0922	0.111
>80	0.126	0.119	0.138	0.101	0.0940	0.107
